# Extracellular Fluid Excess Is Significantly Associated With Coronary Artery Calcification in Patients With Chronic Kidney Disease

**DOI:** 10.1161/JAHA.118.008935

**Published:** 2018-06-30

**Authors:** Seohyun Park, Chan Joo Lee, Jong Hyun Jhee, Hae‐Ryong Yun, Hyoungnae Kim, Su‐Young Jung, Youn Kyung Kee, Chang‐Yun Yoon, Jung Tak Park, Hyeon Chang Kim, Seung Hyeok Han, Shin‐Wook Kang, Sungha Park, Tae‐Hyun Yoo

**Affiliations:** ^1^ Department of Internal Medicine College of Medicine Institute of Kidney Disease Research Yonsei University College of Medicine Seoul Korea; ^2^ Department of Preventive Medicine Yonsei University College of Medicine Seoul Korea; ^3^ Division of Cardiology Cardiovascular Hospital Yonsei University Health System Yonsei University College of Medicine Seoul Korea

**Keywords:** chronic kidney disease, coronary artery calcification, edema, fluid retention, Clinical Studies, Coronary Artery Disease, Atherosclerosis, Risk Factors

## Abstract

**Background:**

Extracellular fluid (ECF) excess is an independent predictor of cardiovascular morbidity in patients undergoing dialysis. This study aimed to investigate the relationship between ECF status, which is affected by renal function, and coronary artery calcification (CAC), which is a marker of cardiovascular disease, in patients with chronic kidney disease (CKD).

**Methods and Results:**

A total of 1741 patients at all stages of pre‐dialysis CKD from the prospective observational cohort of CMERC‐HI (Cardiovascular and Metabolic Disease Etiology Research Center‐High Risk) were analyzed for the association between ECF status and CAC. ECF status was defined as extracellular water‐to‐total body water ratio (ECW/TBW) measured using bioelectrical impedance analysis. ECF excess was defined as ECW/TBW ≥0.390 or ≥0.400 depending on its severity. To define CAC, Agatston coronary artery calcium scores were measured. A total coronary artery calcium score of ≥400 was defined as CAC. The CKD stages were defined according to estimated glomerular filtration rate calculated using the CKD Epidemiology Collaboration equation. ECW/TBW and the proportion of ECF excess increased with progressing CKD stages. Multivariable logistic regression analyses showed that ECW/TBW was independently associated with CAC (per 0.01 increase of ECW/TBW, odds ratio 1.168, 95% confidence interval, 1.079–1.264, *P*<0.001). The adjusted *R* ^2^ for predicting higher coronary artery calcium scores and CAC significantly improved after ECW/TBW was added to conventional factors. This association was further confirmed by net reclassification and integrated discriminant improvements, sensitivity analysis, and subgroup analysis.

**Conclusions:**

ECF status is independently associated with a high risk of CAC in patients with CKD.

**Study Registration:**

URL: https://www.clinicaltrials.gov/. Unique identifier: NCT02003781.

**Clinical Perspective**

**What Is New?**

- Extracellular fluid status deteriorated with declining renal function and was independently associated with a high risk of coronary artery calcification in patients with chronic kidney disease.
- Our large‐scale study is the first to identify extracellular fluid excess as a nontraditional factor associated with coronary artery calcification in patients at all stages of chronic kidney disease.

**What Are the Clinical Implications?**

- Extracellular fluid status may be a contributing factor to the development of coronary artery calcification in patients with chronic kidney disease.
- Our study provides a basis for further investigations to determine the direct effect of extracellular fluid status on the pathogenesis of coronary artery calcification.

## Introduction

An increasing number of studies have demonstrated that cardiovascular disease is the primary cause of morbidity and mortality in chronic kidney disease (CKD).^1^ The increased incidence of cardiovascular events in patients with CKD is related to both traditional and nontraditional cardiovascular risk factors.^2^ Nontraditional cardiovascular risk factors include uremia‐related factors, inflammation, and abnormal metabolism of calcium and phosphate.^3^, ^4^

Extracellular fluid (ECF) excess is a common condition in patients with advanced CKD and undergoing dialysis, and previous studies have shown that ECF excess is associated with all‐cause mortality and cardiovascular morbidity.^5^, ^6^ Although there is abundant evidence that ECF status is associated with adverse clinical outcomes in patients undergoing dialysis, only a few studies have evaluated the association of fluid excess with cardiovascular risk factors or adverse cardiovascular outcomes in patients with CKD.

The clinical assessment of ECF status is relatively difficult because the physical signs of edema are of limited value in diagnosing excess intravascular volume and tissue hydration status.^7^, ^8^ Bioelectrical impedance analysis (BIA) is a method of assessing ECF status by measuring the impedance of the body to applied electric currents of different frequencies.^9^, ^10^ Accumulating evidence suggests that strict BIA‐guided fluid management has a beneficial impact on blood pressure, arterial stiffness, left ventricular hypertrophy, and survival in patients undergoing dialysis.^11^, ^12^

Thus, we conducted this cross‐sectional study to investigate the association between exacerbated ECF status, which occurs with renal function deterioration, and coronary artery calcification (CAC), which is an indicator of cardiovascular disease, in patients at all stages of CKD.

## Methods

The authors declare that all supporting data are available within the article and its online supplementary files.

### Ethics Statement

This study was conducted in accordance with the Declaration of Helsinki, and the study protocol was approved by the institutional review board at Yonsei University Health System Clinical Trial Center. All patients provided written informed consent before entering the study (institutional review board no. 4‐2013‐0581).

### Study Population

The subjects were selected from the CMERC‐HI, registration number for study on https://clinicaltrials.gov/NCT02003781 (Cardiovascular and Metabolic Diseases Etiology Research Center‐High Risk Cohort) study of the Yonsei University Health System between November 2013 and May 2017. CMERC‐HI is an ongoing, nationwide, and prospective cohort study that included patients at a high risk for cardiovascular disease at the Yonsei University Health System, designed to establish an individualized preventive strategy for cardiovascular and cerebrovascular diseases. The following participants were eligible for inclusion in CMERC‐HI: patients with a high risk for hypertension, namely, hypertensive patients with an estimated glomerular filtration rate (eGFR) of ≥60 mL/min per 1.73 m^2^ and target organ damage, or hypertensive patients with eGFR <60 mL/min per 1.73 m^2^; diabetic patients with a random urine albumin‐to‐creatinine ratio of ≥30 mg/g; patients with end‐stage renal disease undergoing dialysis; relatives of patients who have acute myocardial infarction and who were younger than 55 years (for men) or 65 years (for women); patients with asymptomatic atherosclerotic cardiovascular disease (abdominal aorta diameter ≥3 cm or ankle–brachial index <0.9, carotid plaque or carotid intima‐media thickness ≥0.9 mm, asymptomatic old cerebrovascular accident, or >30% stenosis in at least 1 major coronary artery); patients aged >40 years with rheumatic arthritis and taking methotrexate and steroid; patients with atrial fibrillation with CHA2DS2‐VASc score ≥1; and kidney transplant recipients who underwent transplantation >3 months previously. The exclusion criteria were as follows: acute myocardial infarction history (ST‐segment‐elevation myocardial infarction or non–ST‐segment–elevation myocardial infarction) or acute coronary syndrome (unstable angina), symptomatic peripheral artery disease, symptomatic heart failure, life expectancy <6 months or severe noncardiovascular disease (metastatic cancer, sepsis, liver cirrhosis), and pregnancy or breastfeeding.

**Figure 1:**
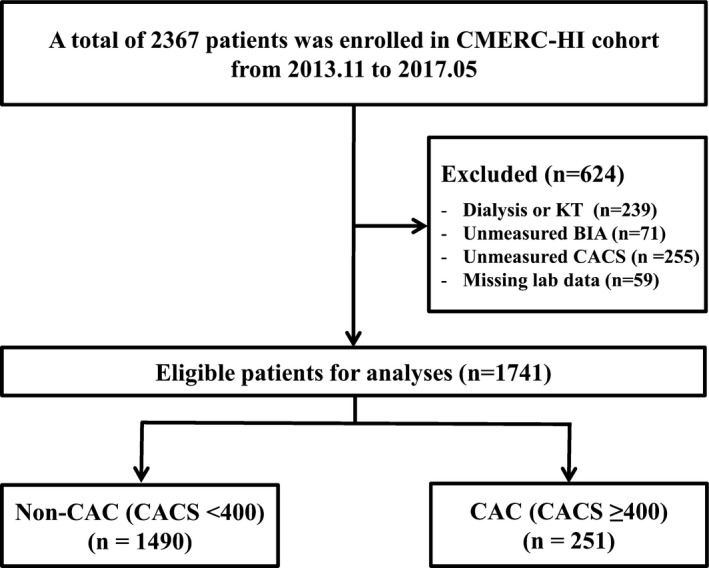
Flow diagram of the study. BIA indicates bioelectrical impedance; CAC, coronary artery calcification; CACS, coronary artery calcium score; CMERC‐HI, Cardiovascular and Metabolic Diseases Etiology Research Center‐High risk; KT, kidney transplantation.

From the total of 2367 patients enrolled in the CMERC‐HI study, we further excluded patients with end‐stage renal disease undergoing dialysis or kidney transplantation, those without BIA data, and those without coronary artery calcium scores (CACSs). Finally, a total of 1741 patients were analyzed for our study (Figure 1).

### Body Composition and ECF Status Measurement

ECF status was assessed with BIA (InBody 720 Body Composition Analyzer; BioSpace, Seoul, Korea) at the time of enrollment. A direct segmental multifrequency BIA method was used with a tetra‐polar 8‐point tactile electrode system, with 30 impedance measurements obtained using 6 frequencies. High‐frequency current passes through the total body water (TBW), whereas low‐frequency current cannot penetrate cell membranes and thus flows exclusively through the extracellular water (ECW). On the basis of a fluid model with these resistances, the ECW, intracellular water, and TBW were calculated. According to the concept that excess ECW results in edema, ECF status was defined as the ECW‐to‐TBW ratio (ECW/TBW), and ECF excess was classified as follows: mild overhydrated state (ECW/TBW 0.390–0.399) and moderate to severe overhydrated state (ECW/TBW ≥0.400) (Biospace Co. Ltd., Seoul, Korea).^13^

### Assessment and Definition of CAC

All examinations were performed with a 320‐row computed tomography system (Aquilion ONE; Toshiba Medical Systems, Tokyo, Japan) with subjects in the supine position on a table, and images were acquired during a single breath hold, to allow image reconstruction in a single cardiac phase. Dual scanograms were used for planning the examination and determining the anatomical range. To obtain the CACSs, a nonenhanced prospective ECG‐gated scan was performed with the following parameters: rotation time, 275 ms; slice collimation, 0.5 mm; slice width, 3.0 mm; tube voltage, 100 kV; and automatic tube current modulation. Images were analyzed in a core workstation with dedicated software (version 4.4.11.82.3430.Beta; TeraRecon, Foster City, CA). Agatston calcium scores were calculated to quantify the extent of CAC. A total CACS of ≥400 was defined as CAC.^14^, ^15^

### Clinical and Biochemical Data Collection

Clinical data and laboratory parameters were collected at the time of cohort enrollment. Diabetes mellitus was defined as a history of diabetes mellitus, use of antidiabetic medications, or fasting plasma glucose levels >126 mg/dL; hypertension was defined as a self‐reported history of hypertension, a history of antihypertensive medication use, or a blood pressure of 140/90 mm Hg or higher at the time of visit; and cardiovascular disease was defined as a composite of coronary occlusive disease, ischemic heart disease, congestive heart failure, atrial fibrillation, atherosclerosis, and cerebrovascular disease. Medications including antihypertensive and antidiabetic drugs and diuretics were investigated based on prescriptions at the time of enrollment. Height, weight, body mass index, and anthropometric data were measured. Moreover, 24‐hour ambulatory blood pressure monitoring was performed using a Takeda TM‐2430 instrument (A&D Medical, Tokyo, Japan). The pulse pressure measured with 24‐hour ambulatory blood pressure monitoring was used in this analysis. Lipid profile components and glucose levels were measured in blood samples obtained after a 12‐hour fast. The eGFR was calculated according to the CKD Epidemiology Collaboration equation based on serum creatinine level.^16^ The CKD stages were defined on the basis of eGFR.

### Statistical Analyses

Continuous variables were expressed as the mean and SD or median with interquartile ranges, and categorical data were presented as counts with percentages. The normality of distribution was ascertained with the Kolmogorov–Smirnov test, Shapiro–Wilk test, and histogram analysis, and skewed continuous parameters were logarithmically transformed before use in parametric procedures. To compare among several groups, analysis of variance with Bonferroni correction or the Kruskal–Wallis test was used for continuous variables, and the χ^2^ test was used for categorical variables. Mantel–Haenszel linear‐by‐linear association method and Jonckheere–Terpstra test were used for analyzing trends. A linear regression analysis was used to identify factors associated with ECF status. To evaluate the independent association of ECF status with CAC, a logistic regression analysis was performed. Factors significantly associated with CAC in the univariate logistic regression analysis (*P*<0.05) were included preferentially in the multivariable logistic analysis. Also, factors independently associated with ECW/TBW in multivariable linear regression analysis were considered as covariates in multivariable logistic analysis. Factors such as serum calcium and phosphate levels and hypertension history were also included in the multivariable model because of its established significance with CAC despite the lack of statistical significance in our results.^17^ An incremental adjustment was performed using the following factors: (1) demographic characteristics including age, sex, waist‐to‐hip ratio, diabetes mellitus, history of cardiovascular disease, smoking status, and pulse pressure; (2) biochemical variables including eGFR, hemoglobin, lipid profiles, albumin, calcium, and phosphate^18^; and (3) use of lipid‐lowering agents, antithrombotic agents, and phosphate binders. We also showed the association of ECF status with CAC in graphical format (cubic spline curves). The predictive value of ECF status for the presence of CAC was determined using receiver operating characteristic curves, net reclassification improvement (NRI), and integrated discrimination improvement (IDI), as previously described.^19^ Risk categories for the categorical NRI were set as 0% to 10% (low), 10% to 20% (middle), and >20% (high) because the patients with coronary artery calcification were 14.4%. For sensitivity analysis, we analyzed 666 patients with NT‐proBNP (amino terminal fragment of the prohormone brain‐type natriuretic peptide) measurements to determine whether fluid overload caused by renal insufficiency, not fluid overload caused by heart failure, was correlated with CAC.

All analyses were performed with SPSS statistical software version 23.0 (IBM Corporation, Armonk, NY), GraphPad Prism version 5.0 (GraphPad Software Inc., San Diego, CA), and R language (version 3.3.1; R Foundation for Statistical Computing) including the smoothHR, pspline, pROC, and PredictABLE packages.

## Results

### Baseline Characteristics

**Figure 2:**
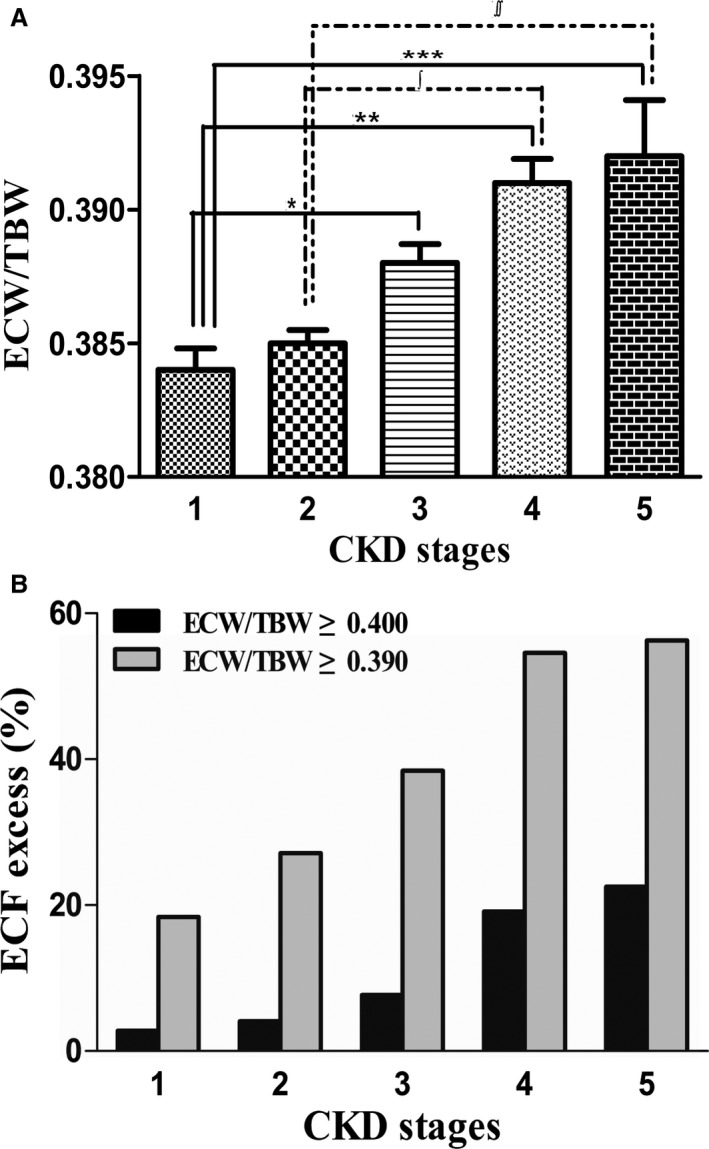
A, Deterioration of extracellular volume status (ECW/TBW) according to stages of CKD. B, Proportion of patients with ECF excess according to renal function: mild‐to‐severe ECF excess (ECW/TBW ≥0.390) and moderate‐to‐severe ECF excess (ECW/TBW ≥0.400); *P=0.003, **P<0.001, ***P=0.001, ∫=0.002, ∫∫=0.0013, as tested by one‐way ANOVA with post hoc Bonferroni correction. CKD indicates chronic kidney disease; ECF, extracellular fluid; ECW/TBW, extracellular water‐to‐total body water ratio.

At the time of enrollment, there was a significant increase in ECF excess with deteriorating renal function among subjects at all stages of CKD. When the subjects were divided into 5 groups according to CKD stage, the results showed that the ECF status was exacerbated and the proportion of ECF excess increased with deteriorating renal function. As evident from the box plots in Figure 2A, the increment of ECW/TBW was more pronounced as the CKD stage became more advanced (from stage 1 to stage 5; 0.384 versus 0.385 versus 0.388 versus 0.391 versus 0.392, *P* for trend <0.001). As CKD progressed, the proportion of patients with ECF excess tended to increase, and the same tendency was observed in patients with mild‐to‐severe overhydrated status (ECW/TBW ≥0.390) and those with moderate‐to‐severe overhydrated status (ECW/TBW ≥0.400) (Figure 2B).

Table 1 lists the characteristics of the 1741 patients according to the quartiles of ECW/TBW. The mean patient age was 60.8 years, and 54.7% patients were men. The median eGFR was 72.2 mL/min per 1.73 m^2^. The mean ECW/TBW was 0.386, and the values according to groups were 0.374, 0.382, 0.388, and 0.400 (first to fourth quartile). The median CACS was 22.0 (0.0–189.3), and 14.4% patients had CAC. Exacerbating ECF status, expressed in quartile range, was significantly associated with CACSs and the percentage of CAC. Baseline characteristics according to CKD stage are shown in Table S1.

**Table 1:** Baseline Characteristics According to Quartiles of ECF Status

	Total (N=1741)	First Quartile (n=434)	Second Quartile (n=437)	Third Quartile (n=433)	Fourth Quartile (n=437)	*P* Value	*P* for Trend^a^
Age, y	60.8±11.4	53.2±11.2	59.0±10.1	62.7±10.5	65.9±9.8	<0.001	<0.001
Men (%)	953 (54.7)	350 (80.6)	258 (59.0)	172 (39.7)	173 (39.6)	<0.001	<0.001
Body mass index, kg/m^2^	25.3±3.8	26.4±4.1	25.1±3.5	24.6±3.4	25.0±3.9	<0.001	<0.001
Waist‐to‐hip ratio	0.93±0.07	0.92±0.06	0.92±0.07	0.92±0.07	0.94±0.07	<0.001	0.001
ECW/TBW	0.386±0.016	0.374±0.010	0.382±0.002	0.388±0.002	0.400±0.024	<0.001	<0.001
Pulse pressure, mm Hg^b^	48.7±22.9	38.5±20.4	39.9±21.8	42.5±22.2	48.7±22.9	<0.001	<0.001
Diabetes mellitus (%)	730 (42.1)	120 (27.8)	141 (32.3)	195 (45.3)	274 (62.8)	<0.001	<0.001
Hypertension (%)	1474 (85.0)	374 (86.6)	363 (83.3)	361 (84.0)	376 (86.2)	0.428	0.959
Cardiovascular disease (%)^c^	750 (43.3)	167 (38.8)	206 (47.4)	201 (46.7)	176 (40.4)	0.019	0.760
Smoking (%)	781 (45.0)	289 (66.8)	211 (48.5)	147 (34.1)	134 (30.8)	<0.001	<0.001
CKD stages (%)						<0.001	<0.001
Stage 1	579 (33.3)	190 (43.7)	163 (37.2)	141 (32.6)	86 (19.7)		
Stage 2	627 (36.0)	152 (34.9)	170 (38.8)	168 (38.8)	138 (31.6)		
Stage 3a	190 (10.9)	43 (9.9)	44 (10.0)	47 (10.9)	56 (12.8)		
Stage 3b	133 (7.6)	26 (6.0)	24 (5.5)	28 (6.5)	55 (12.6)		
Stage 4	141 (8.1)	19 (4.4)	24 (5.5)	29 (6.7)	69 (15.8)		
Stage 5	71 (4.1)	5 (1.1)	13 (3.0)	20 (4.6)	33 (7.6)		
CACS^d^	22.0 (0.0–189.3)	0.0 (0.0–69.5)	9.8 (0.0–126.0)	17.5 (0.0–192.2)	115.6 (11.7–501.5)	<0.001	<0.001
CAC (%)^e^	251 (14.4)	28 (6.5)	40 (9.2)	62 (14.3)	121 (27.7)	<0.001	<0.001
Laboratory findings							
eGFR, mL/min per 1.73 m^2^	72.2±28.6	79.9±25.7	77.2±26.5	72.6±27.7	59.3±29.8	<0.001	<0.001
Hemoglobin, g/dL	13.5±2.0	14.8±1.5	14.0±1.7	13.2±1.7	12.2±1.9	<0.001	<0.001
Total cholesterol, mg/dL	174.1±38.2	181.6±41.7	174.6±35.7	171.7±33.6	168.7±40.0	<0.001	<0.001
LDL, mg/dL	96.2±30.9	101.1±32.3	96.6±30.6	94.6±28.7	92.4±31.3	0.001	<0.001
HDL, mg/dL	49.5±13.5	48.2±12.2	50.6±14.4	49.9±13.0	49.4±14.1	0.086	0.342
Triglyceride, mg/dL	139.2±79.9	161.0±95.2	138.7±79.3	129.1±71.3	128.3±67.2	<0.001	<0.001
Albumin, g/dL	4.2±0.3	4.4±0.3	4.3±0.3	4.2±0.3	4.1±0.4	<0.001	<0.001
Calcium, mg/dL	9.15±0.46	9.24±0.37	9.19±0.45	9.11±0.43	9.05±0.53	<0.001	<0.001
Phosphate, mg/dL	3.63±0.55	3.52±0.50	3.60±0.56	3.68±0.51	3.72±0.60	<0.001	<0.001
Sodium, mmol/L	141.4±2.2	141.1±2.0	141.4±2.1	141.6±2.3	141.5±2.6	0.012	<0.001
Potassium, mmol/L	4.6±0.5	4.5±0.4	4.5±0.5	4.6±0.5	4.6±0.5	0.001	<0.001
Chloride, mmol/L	103.6±3.2	102.8±2.6	103.5±2.9	103.8±2.4	104.6±2.5	<0.001	<0.001
hs‐CRP, mg/L^d^	0.8 (0.5–1.5)	0.9 (0.6–1.7)	0.7 (0.5–1.4)	0.8 (0.5–1.4)	0.8 (0.5–1.7)	0.002	0.016
uACR, mg/gCr^d^	4.8 (1.1–37.9)	3.2 (0.9–34.7)	6.7 (1.0–44.9)	3.8 (0.9–21.3)	9.0 (1.8–63.1)	<0.001	<0.001
Medications							
Antihypertensive drugs^f^	1335 (76.7)	329 (75.8)	325 (74.4)	335 (77.4)	346 (79.2)	0.373	0.154
Lipid‐lowering agents^g^	955 (54.9)	236 (54.4)	229 (52.4)	238 (55.0)	252 (57.7)	0.476	0.259
Diuretics	429 (24.6)	98 (22.5)	100 (22.8)	96 (22.2)	135 (30.9)	0.006	0.008
Antithrombotic agents^h^	667 (38.3)	156 (35.9)	158 (36.2)	167 (38.6)	186 (42.6)	0.155	0.037
Oral calcium	22 (1.3)	2 (0.5)	4 (0.9)	9 (2.1)	7 (1.6)	0.147	0.056
Calcium‐based phosphate binder	6 (0.3)	0 (0.0)	0 (0.0)	3 (0.7)	3 (0.7)	0.110	0.028
Non‐Ca‐based phosphate binder	1 (0.1)	0 (0.0)	0 (0.0)	1 (0.2)	0 (0.0)	0.388	0.656
Phosphate binders^i^	7 (0.4)	0 (0.0)	0 (0.0)	4 (0.9)	3 (0.7)	0.062	0.028

CAC indicates coronary artery calcification; CACS, coronary artery calcium score; CKD, chronic kidney disease; ECF, extracellular fluid; ECF/TBW, extracellular water‐to‐total body water ratio; eGFR, estimated glomerular filtration rate; HDL, high‐density lipoprotein cholesterol; hs‐CRP, high‐sensitivity C‐reactive protein; LDL, low‐density lipoprotein cholesterol; uACR, urine albumin‐to‐creatinine ratio.

a *P* values by the Mantel–Haenszel linear‐by‐linear association method or Jonckheere–Terpstra test.

b Mean value of pulse pressure measured through 24‐h ambulatory blood pressure monitoring.

c Cardiovascular diseases included coronary occlusive disease, ischemic heart disease, congestive heart failure, atrial fibrillation, atherosclerosis, and cerebrovascular disease.

d Kruskal–Wallis test.

e A total CACS of ≥400 was defined as CAC.

f Antihypertensive drugs included angiotensin receptor blockers, angiotensin‐converting enzyme inhibitors, calcium channel blocker, β‐blocker, and α‐blocker.

g Lipid‐lowering agents included statin, fibrate, and nicotinic acid.

h Antithrombotic agents included aspirin, other antiplatelets, and anticoagulants.

i Phosphate binders included both calcium‐based and non‐calcium‐based phosphate binders.

### Association of Clinical and Biochemical Variables With ECF Status

In multivariable linear regression to assess the association of ECW/TBW with variables that were significantly correlated with ECW/TBW in univariable linear regression analysis (Table S2), CACS was independently associated with ECF status after adjusting for multiple confounders. In addition, age, body mass index, hemoglobin level, and albumin level were related to ECW/TBW in multivariable analysis (Table 2).

**Table 2:** Linear Regression Analysis of Variables Associated With ECF Status

	Univariable		Multivariable^a^	
	β (95% CI)	*P* Value	β (95% CI)	*P* Value
Age (per 1 y)	0.029 (0.023–0.036)	<0.001	0.013 (0.003–0.024)	0.009
Men (vs women)	−0.509 (−0.660 to −0.359)	<0.001	−0.095 (−0.421 to 0.231)	0.566
BMI (per 1 kg/m^2^)	−0.043 (−0.063 to −0.023)	<0.001	−0.035 (−0.067 to −0.003)	0.033
Waist‐to‐hip ratio (per 0.01)	0.012 (0.001–0.023)	0.037	0.014 (−0.004 to 0.032)	0.131
Diabetes mellitus	0.431 (0.277–0.583)	<0.001	0.182 (−0.047 to 0.411)	0.119
Smoking history	−0.484 (−0.635 to −0.332)	<0.001	−0.217 (−0.512 to 0.078)	0.148
CACS (per 1 log)^b^	0.349 (0.250–0.453)	<0.001	0.239 (0.089–0.388)	0.002
Pulse pressure (per 1 mm Hg)	0.005 (0.002–0.008)	0.004	−0.001 (−0.006 to 0.004)	0.608
eGFR (per 1 mL/min per 1.73 m^2^)	−0.009 (−0.012 to −0.006)	<0.001	0.002 (−0.002 to 0.007)	0.351
Hemoglobin (per 1 g/dL)	−0.254 (−0.293 to −0.215)	<0.001	−0.143 (−0.218 to −0.068)	<0.001
Total cholesterol (per 1 mg/dL)	−0.002 (−0.004 to 0.000)	0.024	0.001 (−0.002 to 0.003)	0.718
Albumin (per 1 g/dL)	−1.073 (−1.292 to −0.853)	<0.001	−0.636 (−1.022 to −0.249)	0.001
Calcium (per 1 mg/dL)	−0.475 (−0.641 to −0.309)	<0.001	−0.182 (−0.429 to 0.065)	0.148
Phosphate (per 1 mg/dL)	0.238 (0.102–0.379)	0.001	−0.030 (−0.231 to 0.171)	0.770
Chloride (per 1 mg/dL)	0.064 (0.037–0.091)	<0.001	0.013 (−0.021 to 0.048)	0.447
uACR (per 1 log)^b^	0.232 (0.021–0.441)	0.031	−0.185 (−0.484 to 0.114)	0.225

BMI indicates body mass index; CACS, coronary artery calcium score; CI, confidence interval; ECF, extracellular fluid; eGFR, estimated glomerular filtration rate; uACR, urine albumin‐to‐creatinine ratio.

a Adjusted for age; sex; BMI; waist‐to‐hip ratio; diabetes mellitus; smoking history; log‐transformed CACS; pulse pressure; eGFR; serum hemoglobin, total cholesterol, albumin, calcium, phosphate, and chloride levels; and log‐transformed urine albumin‐to‐creatinine ratio.

b Log transformed.

### Independent Association of ECF Status With CAC

**Figure 3:**
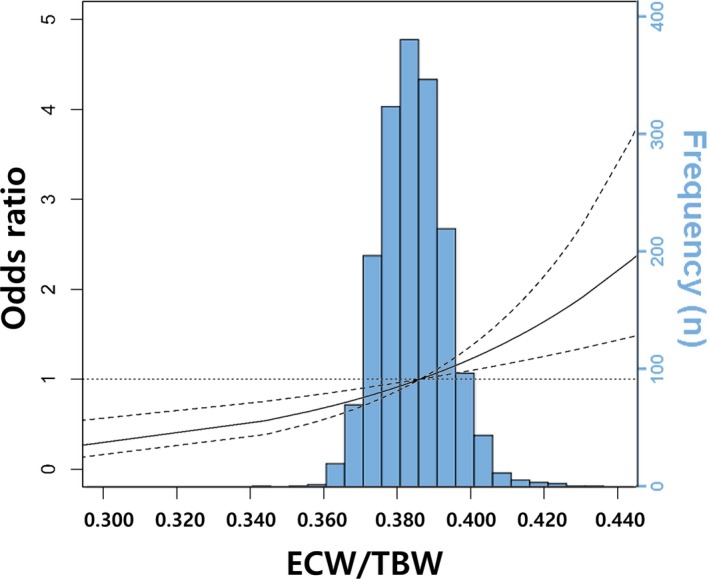
Multivariable adjusted cubic spline plots showing the odds ratio of coronary artery calcification (95% confidence intervals) by extracellular fluid status. The solid line represents the odds ratio and the dashed lines represent the 95% confidence intervals. The model is adjusted for age, sex, waist‐to‐hip ratio, hypertension, diabetes mellitus, cardiovascular disease, smoking status, pulse pressure, hemoglobin level, lipid profiles, albumin level, estimated glomerular filtration rate, calcium level, phosphate level, and use of lipid‐lowering agents, antithrombotic agents, and phosphate binders. The superimposed blue‐colored histogram shows the distribution of extracellular fluid status in the study. ECW/TBW indicates extracellular water‐to‐total body water ratio.

ECF status had a statistically significant association with CAC in all analyses including univariable and stepwise, adjusted for demographic characteristics, laboratory variables, and medication history (model 3 in Table 3; per 0.01 increase, odds ratio 1.168, 95% confidence interval [CI], 1.079–1.264, *P*<0.001). Cubic spline plots showed that the risk of CAC increased steadily with exacerbating ECF status (Figure 3). Significant variables in the univariable analysis (Table S3) and mineral parameters such as calcium and phosphate, which are already known risk factors for CAC, were included in the multivariable models.

**Table 3:** Unadjusted and Adjusted ORs of ECF Status for the Presence of CAC^a^

	OR	95% CI	*P* Value
ECW/TBW (per 0.01 increase)	1.297	1.147 to 1.467	<0.001
Model 1^b^	1.184	1.080 to 1.298	<0.001
Model 2^c^	1.162	1.073 to 1.258	<0.001
Model 3^d^	1.168	1.079 to 1.264	<0.001

CAC indicates coronary artery calcification; CI, confidence interval; ECF, extracellular fluid; ECW/TBW, extracellular water‐to‐total body water ratio; ORs, odds ratios.

a A total Agatston CAC score of ≥400 was defined as CAC.

b Adjusted for age, sex, waist‐to‐hip ratio, hypertension, diabetes mellitus, cardiovascular disease, smoking status, and pulse pressure.

c Adjusted for model 1+estimated glomerular filtration rate, hemoglobin, low‐density lipoprotein cholesterol, albumin, calcium, and phosphate.

d Adjusted for model 2+use of lipid‐lowering agents, antithrombotic agents, and phosphate binders.

### Relative Contribution and Discrimination Ability of ECF Status for CAC

**Figure 4:**
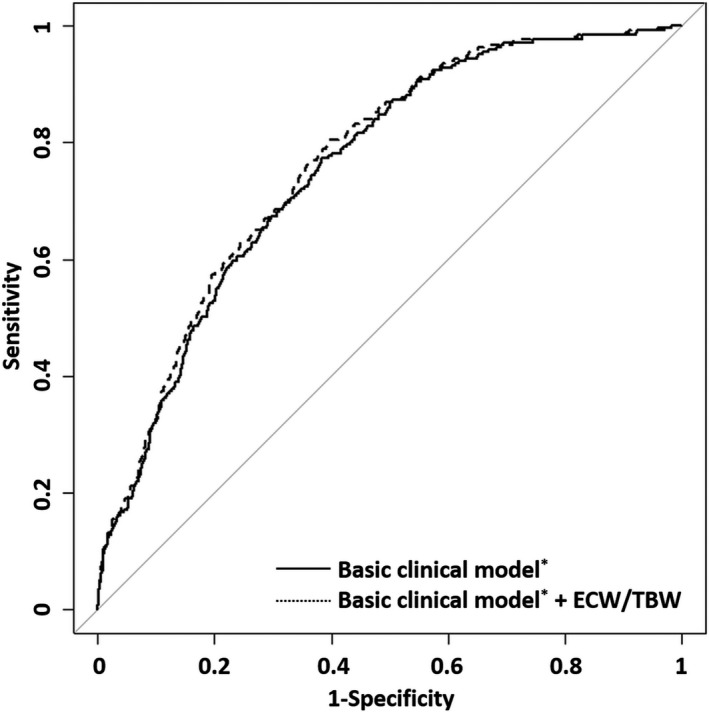
Receiver operating characteristic analysis for the presence of coronary artery calcification. *Adjusted for age; sex; waist‐to‐hip ratio; hypertension; diabetes mellitus; cardiovascular disease; smoking status; pulse pressure; estimated glomerular filtration rate; hemoglobin, low‐density lipoprotein cholesterol, albumin, calcium, and phosphate levels; and use of lipid‐lowering agents, antithrombotic agents, and phosphate binders ECW/TBW, extracellular water‐to‐total body water ratio.

We then evaluated whether ECF status can contribute to the presence of CAC through ROC curve analysis. The areas under the receiver operating characteristic curve for the presence of CAC increased when ECW/TBW was added to the basic clinical model consisting of age; sex; waist‐to‐hip ratio; hypertension; diabetes mellitus; cardiovascular disease; smoking status; pulse pressure; eGFR; hemoglobin, low‐density lipoprotein cholesterol, albumin, calcium, and phosphate levels; and use of lipid‐lowering agents, antithrombotic agents, and phosphate binders (0.757 versus 0.767, *P*=0.001) (Figure 4).

To confirm the impact of ECF status on the predictive power for the presence of CAC, we carried out reclassification analyses. Adding ECW/TBW to the basic clinical model significantly improved NRI (categorical NRI=0.051, *P*=0.004; continuous NRI=0.571, *P*<0.001) and IDI (IDI=0.010, *P*<0.001) (Table 4 and Table S4). This finding suggested that ECF status improved the predictive power for the presence of CAC.

**Table 4:** Reclassification and Discrimination Improvement for the Presence of CAC^a^ According to ECF Status

	Presence of CAC	
	Values	*P* Value
AUC (95% CI)		
Basic clinical model^b^	0.757 (0.728–0.787)	
Basic clinical model^b^+ECW/TBW	0.767 (0.738–0.796)	<0.001^c^
NRI (95% CI)		
Categorical	0.051 (0.016–0.087)	0.004^c^
Continuous	0.571 (0.441–0.701)	<0.001
IDI (95% CI)	0.010 (0.005–0.015)	<0.001^c^

AUC indicates area under the receiver operating characteristic curve; CAC, coronary artery calcification; CI, confidence interval; ECF, extracellular fluid; ECW/TBW, extracellular water‐to‐total body water ratio; IDI, integrated discrimination improvement; NRI, net reclassification improvement.

a A total Agatston CAC score of ≥400 was defined as CAC.

b Adjusted for age; sex; waist‐to‐hip ratio; hypertension; diabetes mellitus; cardiovascular disease; smoking status; pulse pressure; estimated glomerular filtration rate; hemoglobin, low‐density lipoprotein cholesterol, albumin, calcium, and phosphate levels; and use of lipid‐lowering agents, antithrombotic agents, and phosphate binders.

c *P* value for the improvement of predictive power of basic clinical model+ECW/TBW compared with the basic clinical model.

### Sensitivity Analyses

For sensitivity analyses, we further analyzed the association between ECF and CACSs as well as the presence of CAC. In univariate and multivariate analyses, ECF status was statistically significantly associated with log‐transformed CACSs (model 3 in Table S5; per 0.01 increase, β=0.040, 95% CI, 0.019–0.061, *P*<0.001).

In consideration of the possibility that ECF status was exacerbated by cardiac dysfunction rather than renal impairment, we performed sensitivity analysis of 666 patients with NT‐proBNP measurements. As with the analysis results thus far, ECF status showed a strong association with CAC (CAC=83 [12.5%], per 0.01 increase, fully adjusted odds ratio 2.178, 95% CI, 1.412–3.361, *P*<0.001) (Table S6).

### Subgroup Analyses

We further evaluated the association between ECF status and CAC in subgroups. We divided the whole cohort into 3 groups: hypertension; diabetes mellitus with albuminuria; and cardiovascular disease consisting of asymptomatic peripheral vascular disease, atrial fibrillation, and heart failure. This association remained unaltered in the group with hypertension (CAC=65 [8.8%], per 0.01 increase of ECW/TBW, fully adjusted odds ratio 2.036, 95% CI, 1.271–3.261, *P*=0.003) and the group with diabetes mellitus with albuminuria (CAC=152 [20.8%], per 0.01 increase, fully adjusted odds ratio 1.867, 95% CI, 1.409–2.4763, *P*<0.001). In the group with cardiovascular disease, ECF status was also associated with CAC, but not statistically significantly (Table S7).

We performed additional subgroup analyses. To determine whether the results of this study are maintained regardless of CKD severity, we divided the subjects into 2 categories: early CKD group consisting of patients with CKD stage 1 to 2 and advanced CKD group consisting of patients with CKD 3 to 5. The results according to the severity of CKD were similar to our main outcomes, and the association was stronger in the advanced CKD group (Table S8). Finally, we conducted another subgroup analysis according to the use of diuretics. Although the use of diuretics was not associated with ECF status in the linear regression analyses (Table S2) and there was no interaction effect between diuretics and ECF status (data not shown), it was already known that diuretics affect the ECF status. From the analysis, we found that the results were consistent with our primary analysis and the association was more intensified in the group of diuretics users (Table S9).

## Discussion

In the present study, we investigated for the first time the association of ECF status with CAC. Preferentially, ECF status was exacerbated and the proportions of patients with ECF excess increased as renal function declined in patients at all stages of CKD. Moreover, the exacerbation of ECF status with declining renal function was independently related to CAC. The association maintained its significance after extensive adjustments for confounding factors in the statistical models to evaluate the predictive power of ECF status for CAC, and in the subgroup and sensitivity analyses. The consistent results across a series of analyses indicate that our findings are robust.

CACS is considered an index of the severity of CAC and is associated with an increased risk for coronary heart disease events, even when other risk factors for coronary heart disease are taken into account. The relative risks associated with increasing CACSs are at least as large as those associated with established coronary heart disease risk factors in the general population.^20^ Studies of CACSs in patients with CKD are limited. The incidence of CAC and the CACSs in patients with CKD are higher than those in the general population and lower than those in patients undergoing dialysis.^21^ The Chronic Renal Insufficiency Cohort study reported a graded relationship between the severity of CKD and CAC (or CACSs).^22^ Although there is controversy about whether CAC has a diagnostic or prognostic value for coronary artery disease in patients with CKD, positive results have been reported recently. The studies of Yiu et al^23^ and Haydar et al^24^ showed that CACSs predict coronary artery disease in patients with CKD and in the general population, and Russo et al^25^ found that patients with CACS >100 had a greater incidence of cardiac events than patients with CACS ≤100, suggesting that CAC defined as CACSs is an independent predictor of cardiovascular disease in patients with CKD.

The possible explanation for why ECF excess causes arterial calcification in patients with CKD may be derived from the mechanical biology or mechanotransduction of vascular cells. In the presence of ECF excess, urinary excretion of sodium and fluid is increased through a negative feedback mechanism to maintain fluid homeostasis. However, if renal function is impaired, urinary excretion of sodium and fluid is reduced and the intrarenal renin–angiotensin–aldosterone system is triggered, leading to persistent and exacerbated ECF excess.^26^, ^27^ This persistent ECF excess results in pathologic mechanical stimuli on vascular endothelial and smooth muscle cells (VSMCs). In endothelial cells, the biological response of this circumferential stretch promotes the release of angiotensin II and the activation of angiotensin II receptor 1, thereby enhancing superoxide production and reducing the bioavailability of nitric oxide.^28^, ^29^ This results in low, oscillating, or reversing shear stress, which in turn leads to atherosclerosis and vascular calcification.^30^, ^31^ VSMCs sense the disturbed wall shear stress and pressure distension caused by intravascular and interstitial fluid overload through membrane mechanoreceptors, and activate the signaling pathways such as small guanosine triphosphatase related to Ras A (RhoA)/Rho‐associated protein kinase, mitogen‐activated protein kinase, phosphatidylinositol‐3‐kinase/protein kinase B, and extracellular signal‐regulated kinase.^32^, ^33^, ^34^ These signaling pathways induce the proliferation, migration, apoptosis, and osteoblastic differentiation of VSMCs, and altered VSMCs play a major role in vascular calcification.^35^, ^36^ Recent studies have shown that high shear stress induces apoptosis of VSMCs through nitric oxide released by endothelial cells,^37^ and low shear stress upregulates the proliferation and migration of VSMCs via platelet‐derived growth factor and transforming growth factor secreted by endothelial cells.^38^ Therefore, ECF excess may also be involved in vascular calcification through the cross‐talk between endothelial cells and VSMCs. In addition to mechanical stimuli, ECF excess can contribute to vascular calcification through inflammation and oxidation. Several experimental and clinical studies have reported that inflammatory biomarkers such as tumor necrosis factor‐α, IL‐6, vascular adhesion molecule‐1, macrophage, and thrombomodulin were systemically or locally increased in the presence of ECF excess.^39^, ^40^ ECF excess directly induces a phenotype change in VSCMs and indirectly induces oxidative stress, which increases the production of bone morphogenetic protein 2 and endothelial cell–derived microparticles in endothelial cells, thereby enhancing the osteogenic signal acting on VSMCs.^41^, ^42^, ^43^

Among the causes of vascular calcification in patients with impaired renal function, abnormalities in mineral parameters such as calcium and phosphate are a major concern. However, in this study, no relationship was found between phosphate and calcium levels and the presence of CAC. This association is probably more evident in patients undergoing dialysis than in patients with CKD. There are fewer reports on patients with chronic renal failure, and the results are contradictory. Some studies have reported that calcium, phosphate, and parathyroid hormone, as well as dialysis, promote CAC; however, other studies failed to demonstrate such relationships.^44^ In this study, although the levels of calcium and phosphate tended to deteriorate with the progression of renal failure, they were in the normal range suggested by clinical guidelines,^45^, ^46^ even in patients with advanced CKD (Table S1), and we failed to identify the association between mineral parameters and the presence of CAC (data not shown). Despite some studies showing that phosphate level increases in CAC within the normal range,^47^ the Kidney Disease: Improving Global Outcomes and Kidney Disease Outcomes Quality Initiative guidelines do not recommend phosphate lowering in patients with CAC in the normal range.^45^, ^46^ Further research is needed to establish strong evidence.

This study has distinct strengths. This is the first study to identify ECF excess as a nontraditional factor associated with CAC in patients at all stages of CKD. In addition, this study was conducted in a prospective manner and with a large‐scale cohort including 1741 patients. All measurements, including ECF status and CACS, were taken in almost all patients and were performed by a well‐trained evaluator using a standardized protocol in the same machine for each metric. Therefore, these accurate measurements made our findings highly reliable. This study also has some important limitations. First, because of its cross‐sectional design, we could not determine the causative relationship of ECF excess to CAC. Therefore, prospective and longitudinal studies are needed to determine whether ECF excess predicts future CAC. In addition, we expect to be able to confirm this issue upon completion of the ongoing CMERC‐HI study. We also look forward to studying whether ECF status can predict the incidence of CAC in patients without CAC at study enrollment. Second, this study cannot be generalized to the whole CKD population because we used data from a cohort comprising patients at a high risk for cardiovascular disease. However, we cautiously assumed that our findings would be maintained in other CKD populations because we found consistent results in all subgroup and sensitivity analyses. Third, in this study, we used BIA to assess volume status and did not directly measure body water. The criterion standard for assessing fluid status is the tracer dilution technique. However, it is difficult to use in clinical practice and it is known to correlate well with BIA.^48^ Furthermore, the reproducibility and reliability of our multifrequency BIA method have already been verified in previous studies.^49^, ^50^ Fourth, there are unmeasured confounding factors that may alter the relationship between ECW/TBW and CAC. We could not measure the degree of sodium intake associated with ECF status and the levels of parathyroid hormone or vitamin D, known to affect vascular calcification,^51^ at the time of enrollment. Fifth, the mechanism by which ECF excess contributes to vascular calcification is not proven by our own experiments. We have carefully reviewed as much of the literature as possible to make up for these limitations, and we expect further studies to be done.

In conclusion, ECF status, which is affected by renal function, is significantly and independently associated with CAC at all stages of CKD without dialysis. The present study suggests that ECF status may be one of the contributing factors to the development of CAC in patients with CKD and provides a basis for further investigations to determine the direct effect of ECF status on the pathogenesis of CAC.

## Author Contributions

The first author, Seohyun Park, played a key role in the planning of the study, collection of data, derivation and interpretation of the results, and drafting of the article. The 2 corresponding authors, Yoo and Sungha Park, were responsible for reviewing and revising the whole process of the research plan, the results and interpretation of the research, and the writing process of the article. Professor Yoo, who majored in nephrology, and Professor Sungha Park, who majored in cardiology, confirmed the validity of this article in each field. The other co‐authors contributed to the concept of research, data collection, and the analysis and interpretation of results in part. Yoon contributed to the idea of the study; Hyeon Chang Kim contributed to the design of the study; and Lee provided information on several measurements including coronary artery calcium scores. Jhee, Yun, Hyoungnae Kim, Jung, Kee, Jung Tak Park, Han, and Kang contributed to the analysis and interpretation of results through active discussions.

## Sources of Funding

This research was supported by a grant from the Korea Health Technology R&D Project through the Korea Health Industry Development Institute, funded by the Ministry of Health and Welfare, Republic of Korea (grant no. HI13C0715), and supported by the Basic Science Research Program through the National Research Foundation of Korea funded by the Ministry of Science, ICT and Future Planning (NRF‐2015R1A2A2A01007346).

## Disclosures

None.

## Supporting information


**Table S1.** Baseline Characteristics According to CKD Stages
**Table S2.** Univariate Linear Regression Analysis of Variables Associated With Extracellular Fluid Status
**Table S3.** Univariable Logistic Regression Analyses for Presence of Coronary Artery Calcification*
**Table S4.** Reclassification Improvement for Presence of Coronary Artery Calcification* by Extracellular Fluid Status
**Table S5.** Unadjusted and Adjusted Linear Regression Analyses of Extracellular Fluid Status for the Coronary Artery Calcium Scores*
**Table S6.** Association of ECF Status for the Presence of Coronary Artery Calcification* After Additional Adjustment of NT‐proBNP
**Table S7.** Subgroup Analysis
**Table S8.** Unadjusted and Adjusted Odds Ratios of Extracellular Fluid Status for the Presence of Coronary Artery Calcification* Stratified by the Severity of Chronic Kidney Disease
**Table S9.** Association of ECF Status for the Presence of Coronary Artery Calcification* Depending on Diuretic Use
