# Supplementary material for: Extracellular Fluid Excess Is Significantly Associated With Coronary Artery Calcification in Patients With Chronic Kidney Disease
Source: J Am Heart Assoc. 2018 Jun 30;7(13):e008935. doi: 10.1161/JAHA.118.008935 (PMC6064889; doi:10.1161/JAHA.118.008935)
Supplement: Supplementary file 1 — Table S1. Baseline Characteristics According to CKD Stages Table S2. Univariate Linear Regression Analysis of Variables Associated With Extracellular Fluid Status Table S3. Univariable Logistic Regression Analyses for Presence of Coronary Artery Calcification* Table S4. Reclassification Improvement for Presence of Coronary Artery Calcification* by Extracellular Fluid Status Table S5. Unadjusted and Adjusted Linear Regression Analyses of Extracellular Fluid Status for the Coronary Artery Calcium Scores* Table S6. Association of ECF Status for the Presence of Coronary Artery Calcification* After Additional Adjustment of NT‐proBNP Table S7. Subgroup Analysis Table S8. Unadjusted and Adjusted Odds Ratios of Extracellular Fluid Status for the Presence of Coronary Artery Calcification* Stratified by the Severity of Chronic Kidney Disease Table S9. Association of ECF Status for the Presence of Coronary Artery Calcification* Depending on Diuretic Use [file JAH3-7-e008935-s001.pdf]

SUPPLEMENTAL MATERIAL

Table S1. Baseline characteristics according to CKD stages.

	Total (n=1741)	Stage 1 (n=579)	Stage 2 (n=627)	Stage 3 (n=323)	Stage 4 (n=141)	Stage 5 (n=71)	P for trend[‡‡]
**Age (years)**	60.8 ± 11.4	55.9 ± 10.7	63.6 ± 0.4	62.3 ± 11.4	59.3 ± 12.7	57.9 ± 11.7	<0.001
**Men (%)**	953 (54.7)	300 (51.8)	359 (57.3)	192 (59.4)	71 (50.4)	31 (43.7)	0.866
**Body mass index (kg/m^2)**	25.3 ± 3.8	25.4 ± 4.2	25.5 ± 3.4	25.3 ± 3.8	24.8 ± 4.1	23.6 ± 3.4	0.017
**Waist to hip ratio**	0.93 ± 0.07	0.92 ± 0.06	0.93 ± 0.07	0.94 ± 0.07	0.93 ± 0.08	0.92 ± 0.08	<0.001
**ECW/TBW**	0.386 ± 0.016	0.384 ± 0.020	0.385 ± 0.014	0.388 ± 0.013	0.391 ± 0.018	0.392 ± 0.018	<0.001
**ECW/TBW ≥0.390**	518 (29.8)	107 (27.1)	170 (27.1)	124 (38.4)	77 (54.6)	40 (56.3)	<0.001
**ECW/TBW ≥0.400**	110 (6.3)	16 (2.8)	26 (4.1)	25 (7.7)	27 (19.1)	16 (22.5)	<0.001
**Pulse pressure (mmHg)** [*]	48.7 ± 22.9	38.6 ± 21.3	42.0 ± 22.8	44.4 ± 21.8	49.7 ± 21.6	53.7 ± 18.8	<0.001
**Diabetes (%)**	730 (42.1)	214 (37.1)	266 (42.8)	160 (49.5)	63 (44.7)	27 (28.6)	0.019
**Hypertension (%)**	1474 (85.0)	460 (79.7)	541 (87.0)	288 (89.2)	124 (87.9)	61 (85.9)	0.001
**Cardiovascular disease (%)**[‡]	750 (43.3)	281 (48.7)	341 (54.8)	95 (29.4)	22 (15.7)	11 (15.9)	<0.001
**Smoking (%)**	781 (45.0)	249 (43.2)	287 (45.8)	163 (50.8)	57 (40.7)	25 (35.2)	0.871
**CACS** [†]	22.0 (0.0 – 189.3)	0.0 (0.0 – 80.5)	43.7 (0.0 – 256.5)	45.2 (0.0 – 267.0)	42.1 (0.0 – 255.7)	20.1 (0.0 – 132.0)	<0.001
**CAC (%)**[§]	251 (14.4)	41 (7.1)	104 (16.6)	68 (21.1)	24 (17.0)	14 (19.7)	<0.001
**Laboratory findings**							
eGFR (mL•min-1•1.73 m^{-2})	72.2 ± 28.6	100.6 ± 7.9	77.4 ± 8.7	46.5 ± 8.8	22.7 ± 4.3	10.8 ± 2.7	<0.001
Hemoglobin (g/dL)	13.5 ± 2.0	14.3 ± 1.5	14.0 ± 1.7	13.2 ± 2.0	11.3 ± 1.3	10.4 ± 1.4	<0.001
Total cholesterol (mg/dL)	174.1 ± 38.2	176.5 ± 38.8	172.3 ± 34.0	173.7 ± 41.7	177.8 ± 43.6	166.5 ± 38.8	0.070
LDL (mg/dL)	96.2 ± 30.9	98.2 ± 32.2	94.8 ± 29.3	94.3 ± 29.8	100.5 ± 34.8	90.4 ± 19.2	0.165
HDL (mg/dL)	49.5 ± 13.5	51.5 ± 13.5	50.8 ± 12.6	48.1 ± 14.5	45.5 ± 12.7	41.5 ± 12.7	<0.001
Triglyceride (mg/dL)	139.2 ± 79.9	134.7 ± 79.4	136.9 ± 76.7	148.5 ± 92.0	144.7 ± 63.3	146.2 ± 80.5	<0.001
Albumin (g/dL)	4.2 ± 0.3	4.3 ± 0.3	4.3 ± 0.3	4.2 ± 0.4	4.0 ± 0.4	3.9 ± 0.4	<0.001
Calcium (mg/dL)	9.15 ± 0.46	9.17 ± 0.38	9.22 ± 0.42	9.16 ± 0.46	8.95 ± 0.54	8.65 ± 0.68	<0.001
Phosphate (mg/dL)	3.63 ± 0.55	3.61 ± 0.48	3.52 ± 0.50	3.60 ± 0.52	3.86 ± 0.51	4.38 ± 0.93	<0.001
Sodium (mmol/L)	141.4 ± 2.2	141.2 ± 2.2	141.5 ± 2.2	141.6 ± 2.4	141.5 ± 2.4	140.9 ± 3.1	0.028
Potassium (mmol/L)	4.6 ± 0.5	4.4 ± 0.4	4.5 ± 0.4	4.7 ± 0.5	5.0 ± 0.5	4.9 ± 0.5	<0.001
Chloride (mmol/L)	105.3 ± 4.8	102.7 ± 2.7	103.1 ± 2.5	104.3 ± 3.4	106.5 ± 3.3	105.3 ± 4.8	<0.001

hs-CRP (mg/L)†	0.8 (0.5 – 1.5)	0.8 (0.5 – 1.5)	0.8 (0.5 – 1.4)	0.9 (0.6 – 1.7)	0.8 (0.5 – 2.3)	0.8 (0.5 – 1.8)	0.016
uACR (mg/gCr)†	4.8 (1.1 – 37.9)	1.5 (0.7 – 7.2)	2.6 (0.9 – 9.5)	14.2 (3.1 – 57.8)	64.8 (26.5 – 154.0)	82.2 (36.8 – 186.2)	<0.001
**Medications**							
Antihypertensive drugs‖	1335 (76.7)	407 (70.3)	483 (77.0)	261 (80.8)	122 (86.5)	62 (87.3)	<0.001
Lipid lowering agents#	955 (54.9)	319 (55.1)	384 (61.2)	156 (48.3)	65 (46.1)	31 (43.7)	0.002
Diuretics	429 (24.6)	109 (18.8)	173 (27.6)	80 (24.8)	42 (29.8)	25 (35.2)	<0.001
Antithrombotic agents**	667 (38.3)	199 (34.4)	284 (45.3)	114 (35.3)	47 (33.3)	23 (32.4)	0.540
Oral calcium	22 (1.3)	0 (0.0)	5 (0.8)	6 (1.9)	3 (2.1)	8 (11.3)	<0.001
Calcium-based phosphate binder	6 (0.3)	0 (0.0)	0 (0.0)	1 (0.3)	2 (1.4)	3 (4.2)	<0.001
Non-Ca-based phosphate binder	1 (0.1)	0 (0.0)	0 (0.0)	1 (0.3)	0 (0.0)	0 (0.0)	0.429
Phosphate binders∫	7 (0.4)	0 (0.0)	0 (0.0)	2 (0.6)	2 (1.4)	3 (4.2)	<0.001

*Mean value of pulse pressure measured by 24-hour ambulatory blood pressure monitoring, †Kruskal-Wallis test, ‡Cardiovascular diseases included coronary occlusive disease, ischemic heart disease, congestive heart failure, arrhythmia, atherosclerosis, and cerebrovascular disease., §A total CACS ≥ 400 was defined as CAC., ‖ Antihypertensive drugs included angiotensin receptor blockers, angiotensin-converting enzyme inhibitors, calcium channel blocker, β-blocker, and α-blocker., # Lipid lowering agents included statin, fibrate, and nicotinic acid., **Antithrombotic agents included aspirin, other anti-platelets and anti-coagulants., ∫ Phosphate binders included both calcium-based and non-calcium-based phosphate binders, and ‡‡P values by the Mantel–Haenszel's linear by linear association method or Jonckheere-Terpstra test

ECF/TBW, extracellular water to total body water ratio; CKD, chronic kidney disease; CACS, coronary artery calcium score; CAC, coronary artery calcification; eGFR, estimated glomerular filtration rate; LDL, low density lipoprotein cholesterol; HDL, high density lipoprotein cholesterol; hs-CRP, high-sensitivity C-reactive protein; uACR, urine albumin to creatinine ratio

Table S2. Univariate linear regression analysis of variables associated with extracellular fluid status.

	β	95% CI	*P*
**Age (per 1 year)**	0.029	0.023 – 0.036	<0.001
**Men (vs. Women)**	-0.509	-0.660 – -0.359	<0.001
**Body mass index (per 1 kg/m^2)**	-0.043	-0.063 – -0.023	<0.001
**Waist to hip ratio (per 0.01)**	0.012	0.001 – 0.023	0.037
**Pulse pressure (per 1 mmHg)**[†]	0.005	0.002 – 0.008	0.004
**Hypertension**	-0.026	-0.238 – 0.189	0.815
**Diabetes**	0.431	0.277 – 0.583	<0.001
**Cardiovascular diseases** [‡]	-0.046	-0.197 – 0.108	0.556
**Smoking history**	-0.484	-0.635 – -0.332	<0.001
**CACS (per 1 log)** [*]	0.352	0.250 – 0.453	<0.001
**Laboratory findings**			
eGFR (per 1 mL•min-1•1.73 m^{-2})	-0.009	-0.012 – -0.006	<0.001
Hemoglobin (per 1 g/dL)	-0.254	-0.293 – -0.215	<0.001
Total cholesterol (per 1 mg/dL)	-0.002	-0.004 – 0.000	0.024
LDL (per 1 mg/dL)	-0.002	-0.004 – 0.001	0.157
HDL (per 1 mg/dL)	0.003	-0.003 – 0.009	0.355
Triglyceride (per 1 mg/dL)	-0.002	-0.003 – -0.001	<0.001
Albumin (per 1 g/dL)	-1.073	-1.292 – -0.853	<0.001
Calcium (per 1 mg/dL)	-0.475	-0.641 – -0.309	<0.001
Phosphate (per 1 mg/dL)	0.238	0.102 – 0.379	0.001
Sodium	0.032	-0.005 – 0.069	0.091
Potassium	0.177	-0.002 – 0.358	0.054
Chloride	0.064	0.037 – 0.091	<0.001
hs-CRP (per 1 log) [*]	0.224	-0.493 – 0.960	0.544
uACR (per 1og) [*]	0.232	0.021 – 0.441	0.031
**Medications**			
Antihypertensive drugs [§]	-0.132	-0.311 – 0.049	0.150
Lipid lowering agents [‖]	-0.016	-0.168 – 0.138	0.834
Diuretics	-0.161	-0.016 – 0.336	0.073
Antithrombotic agents [#]	0.028	-0.126 – 0.186	0.723
Oral calcium	0.280	-0.401 – 0.960	0.420
Phosphate binders [**]	0.656	-0.545 – 1.857	0.284

[*] Log transformed, [†] Mean value of pulse pressure measured by 24-hour ambulatory blood pressure monitoring, [‡] Cardiovascular diseases included coronary occlusive disease, ischemic heart disease, congestive heart failure, arrhythmia, atherosclerosis, and cerebrovascular disease., [§] Antihypertensive drugs included angiotensin receptor blockers, angiotensin-converting enzyme inhibitors, calcium channel blocker, β-blocker, and α-blocker., [‖] Lipid lowering agents included statin, fibrate, and nicotinic acid., [#] Antithrombotic agents included aspirin, other anti-platelets and anti-coagulants., [**] Phosphate binders included both calcium-based and non-calcium-based phosphate binders.

CI, confidence interval; CACS, coronary artery calcium score; eGFR, estimated glomerular filtration rate; LDL, low density lipoprotein cholesterol; HDL, high density lipoprotein cholesterol; hs-CRP, high-sensitivity C-reactive protein; uACR, urine albumin to creatinine ratio

Table S3. **Univariable logistic regression analyses for presence of coronary artery calcification*.**

	Univariable		
	**OR**	**95% CI**	**P**
**Age (per 1 year)**	1.055	1.040 – 1.070	<0.001
**Men (vs. Women)**	1.944	1.463 – 2.583	<0.001
**Body mass index (per 1 kg/m^2)**	1.000	0.965 – 1.035	0.989
**Waist hip ratio (per 0.01)**	1.054	1.033 – 1.075	<0.001
**Pulse pressure (per 1 mmHg)** ‡	1.007	1.000 – 1.013	0.035
**Hypertension**	1.287	0.861 – 1.923	0.218
**Diabetes**	2.459	1.867 – 3.238	<0.001
**Cardiovascular diseases** §	1.315	1.005 – 1.720	0.046
**Smoking history**	1.703	1.299 – 2.231	<0.001
**ECW/TBW (per 0.01)**	1.297	1.147 – 1.467	<0.001
**Laboratory findings**			
eGFR (per 1 mL•min-1•1.73 m^{-2})	0.988	0.983 – 0.992	<0.001
Hemoglobin (per 1 g/dL)	0.879	0.820 – 0.942	<0.001
Total cholesterol (per 1 mg/dL)	0.991	0.987 – 0.995	<0.001
LDL (per 1 mg/dL)	0.988	0.983 – 0.993	<0.001
HDL (per 1 mg/dL)	0.987	0.977 – 0.998	0.021
Triglyceride (per 1 mg/dL)	0.999	0.997 – 1.001	0.411
Albumin (per 1 g/dL)	0.538	0.371 – 0.779	0.001
Calcium (per 1 mg/dL)	1.184	0.884 – 1.586	0.258
Phosphate (per 1 mg/dL)	0.923	0.722 – 1.181	0.524
hs-CRP (per 1 log) †	0.835	0.255 – 3.097	0.788
uACR (per 1og) †	1.273	0.921 – 1.760	0.144
**Medications**			
Antihypertensive drugs ‖	1.232	0.886 – 1.712	0.215
Lipid lowering agents #	1.346	1.025 – 1.769	0.033
Diuretics	1.300	0.966 – 1.750	0.083
Antithrombotic agents **	1.813	1.386 – 2.371	<0.001
Oral calcium	1.324	0.444 – 3.946	0.614
Phosphate binders ʃ	4.494	1.000 – 20.200	0.050

*A total Agatston coronary artery calcium score ≥ 400 was defined as coronary artery calcification., †log transformed, ‡ Mean value of pulse pressure measured by 24-hour ambulatory blood pressure, § Cardiovascular diseases included coronary occlusive disease, ischemic heart disease, congestive heart failure, arrhythmia, atherosclerosis, and cerebrovascular disease., ‖ Antihypertensive drugs included angiotensin receptor blockers, angiotensin-converting enzyme inhibitors, calcium channel blocker, β-blocker, and α-blocker., # Lipid lowering agents included statin, fibrate, and nicotinic acid., ** Antithrombotic agents included aspirin, other anti-platelets and anti-coagulants., ʃ Phosphate binders included both calcium-based and non-calcium-based phosphate binders.

OR, odds ratio; CI, confidence interval; ECW/TBW, extracellular water to total body water ratio; eGFR, estimated glomerular filtration rate; Hb, hemoglobin; LDL, Low density lipoprotein cholesterol; HDL, high density lipoprotein cholesterol; hs-CRP, high-sensitivity C-reactive protein; uACR, urine albumin to creatinine ratio

Table S4. Reclassification improvement for presence of coronary artery calcification[*] by extracellular fluid status.

		Basic clinical model[†] + ECW/TBW			Reclassification	
		<10%	10 – 20%	≥20%	Up	Down
**Non-CAC (CAC <400, n =1490)**						
**Basic clinical model[†]**	<10%	751	11	3	23	64
	10 – 20%	40	373	9		
	≥20%	0	24	279		
**CAC (CACS ≥400, n =251)**						
**Basic clinical model[†]**	<10%	36	2	0	12	6
	10 – 20%	4	63	10		
	≥20%	0	2	134		

[*] A total Agatston coronary artery calcium score ≥ 400 was defined as coronary artery calcification

[†] Adjusted for age, sex, waist to hip ratio, hypertension, diabetes, cardiovascular disease, smoking status, pulse pressure, estimated glomerular filtration rate, hemoglobin, low-density lipoprotein cholesterol, albumin, calcium, phosphate, and use of lipid lowering agents, antithrombotic agents, and phosphate binders

ECW/TBW, extracellular water to total body water ratio; NRI, net reclassification improvement; IDI, integrated discrimination improvement

Table S5. Unadjusted and adjusted linear regression analyses of extracellular fluid status for the coronary artery calcium scores*.

	β	95% CI	*P*
**ECW/TBW (per 0.01 increase)**	0.074	0.052 – 0.095	<0.001
**Model 1**†	0.053	0.032 – 0.073	<0.001
**Model 2** ‡	0.038	0.017 – 0.060	<0.001
**Model 3** §	0.040	0.019 – 0.061	<0.001

* Log transformed coronary artery calcium scores

† Adjusted for age, sex, waist to hip ratio, hypertension, diabetes, cardiovascular disease, smoking status, and pulse pressure

‡ Adjusted for model 1 + estimated glomerular filtration rate, hemoglobin, low-density lipoprotein cholesterol, albumin, calcium, and phosphate

§ Adjusted for model 2 + use of lipid lowering agents, antithrombotic agents, and phosphate binders

ECW/TBW, extracellular water to total body water ratio; OR, odds ratio; CI, confidence interval

Table S6. Association of ECF status for the presence of coronary artery calcification[*] after additional adjustment of NT-proBNP.

	OR	95% CI	P
**ECW/TBW (per 0.01 increase)**	1.941	1.474 – 2.554	<0.001
**Model 1**[†]	1.530	1.099 – 2.129	0.012
**Model 2**[‡]	1.645	1.113 – 2.432	0.013
**Model 3**[§]	1.690	1.139 – 2.507	0.009

[*] A total Agatston coronary artery calcium score ≥ 400 was defined as coronary artery calcification
[†] Adjusted for age, sex, waist to hip ratio, hypertension, diabetes, cardiovascular disease, smoking status, and pulse pressure
[‡] Adjusted for model 1 + estimated glomerular filtration rate, hemoglobin, low-density lipoprotein cholesterol, albumin, calcium, phosphate, and log transformed NT-proBNP
[§] Adjusted for model 2 + use of lipid lowering agents. antithrombotic agents, and phosphate binders

ECW/TBW, extracellular water to total body water ratio; OR, odds ratio; CI, confidence interval

Table S7. Subgroup analysis.

	Hypertension group (CAC/total N=65/767)			DM with albuminuria group (CAC/total N=152/730)			CVD group* (CAC/total N=8/95)		
	**OR**	**95% CI**	**P**	**OR**	**95% CI**	**P**	**OR**	**95% CI**	**P**
**ECW/TBW (per 0.01)**	1.589	1.212 – 2.083	0.001	1.794	1.476 – 2.181	<0.001	0.795	0.273 – 2.313	0.673
**Model 1**[†]	1.355	0.967 – 1.900	0.078	1.821	1.469 – 2.256	<0.001	1.284	0.322 – 5.123	0.724
**Model 2** [‡]	1.875	1.183 – 2.971	0.007	1.869	1.411 – 2.476	<0.001	2.183	0.398 – 11.981	0.369
**Model 3** [§]	2.036	1.271 – 3.261	0.003	1.867	1.409 – 2.473	<0.001	2.843	0.383 – 21.115	0.307

[*] CVD group include asymptomatic peripheral vascular disease, atrial fibrillation, and heart failure patients without diabetes.
[†] Adjusted for age, sex, waist to hip ratio, hypertension, diabetes, cardiovascular disease, smoking status, and pulse pressure
[‡] Adjusted for model 1 + hemoglobin, lipid profiles, albumin, and estimated glomerular filtration rate
[§] Adjusted for model 2 + use of lipid lowering agents and antithrombotic agents

DM, diabetes mellitus; CVD, cardiovascular disease; ECW/TBW, extracellular water to total body water ratio; OR, odds ratio; CI, confidence interval

Table S8. Unadjusted and adjusted odds ratios of extracellular fluid status for the presence of coronary artery calcification* stratified by the severity of chronic kidney disease.

	CKD stage 12 (n = 145/1206)			CKD stage 3-5 (n = 106/535)		
	OR	95% CI	P	OR	95% CI	P
**ECW/TBW (per 0.01 increase)**	1.094	1.014 – 1.182	0.021	1.820	1.475 – 2.246	<0.001
**Model 1†**	1.098	1.014 – 1.188	0.021	1.722	1.402 – 2.240	<0.001
**Model 2 ‡**	1.109	1.021 – 1.205	0.014	2.166	1.580 – 2.970	<0.001
**Model 3 §**	1.113	1.024 – 1.209	0.011	2.238	1.627 – 3.078	<0.001

* A total Agatston coronary artery calcium score ≥ 400 was defined as coronary artery calcification
† Adjusted for age, sex, waist to hip ratio, hypertension, diabetes, cardiovascular disease, smoking status, and pulse pressure
‡ Adjusted for model 1 + estimated glomerular filtration rate, hemoglobin, low-density lipoprotein cholesterol, albumin, calcium, and phosphate
§ Adjusted for model 2 + use of lipid lowering agents, antithrombotic agents, and phosphate binders

ECW/TBW, extracellular water to total body water ratio; OR, odds ratio; CI, confidence interval

Table S9. Association of ECF status for the presence of coronary artery calcification[*] depending on diuretic use.

	Diuretics non-user			Diuretics user		
	**OR**	**95% CI**	**P**	**OR**	**95% CI**	**P**
**ECW/TBW (per 0.01 increase)**	1.171	1.053 – 1.304	0.004	1.643	1.321 – 2.043	<0.001
**Model 1[†]**	1.143	1.054 – 1.239	0.001	1.474	1.156 – 1.878	0.002
**Model 2[‡]**	1.135	1.051 – 1.225	0.001	2.024	1.365 – 3.002	<0.001
**Model 3[§]**	1.137	1.054 – 1.228	0.001	2.232	1.478 – 3.371	<0.001

[*] A total Agatston coronary artery calcium score ≥ 400 was defined as coronary artery calcification
[†] Adjusted for age, sex, waist to hip ratio, hypertension, diabetes, cardiovascular disease, smoking status, and pulse pressure
[‡] Adjusted for model 1 + estimated glomerular filtration rate, hemoglobin, low-density lipoprotein cholesterol, albumin, calcium, and phosphate
[§] Adjusted for model 2 + use of lipid lowering agents, antithrombotic agents, and phosphate binders

ECW/TBW, extracellular water to total body water ratio; OR, odds ratio; CI, confidence interval
